# Editorial: Changes in metabolic processes affecting brain development

**DOI:** 10.3389/fnins.2022.1002010

**Published:** 2022-09-06

**Authors:** Gregory W. Kirschen, Shaoyu Ge

**Affiliations:** ^1^Department of Gynecology and Obstetrics, The Johns Hopkins Hospital, Baltimore, MD, United States; ^2^Department of Neurobiology and Behavior, Stony Brook University, Stony Brook, NY, United States

**Keywords:** oligodendrocytes, TORCH, brain organoids, short chain fatty acids, one carbon metabolism, gut microbiome, neurodevelopment

The developing human brain has its own intrinsic chemistry and metabolic functions dictated by genetic factors and nutrient availability to provide the energy for vast proliferation, differentiation, and migration of neurons and glia, synapse formation, and circuitry establishment. These intrinsic processes interact with and are influenced by the outside world to which the brain will be trained to generate patterns of responses and purposeful behavior. Early life exposures, whether to medications, illicit substances, pathogens, hypoxia, light, or inflammation, all indelibly shape the metabolic and electrical systems that will result in either proper or pathological brain functioning. This special issue which contains original research articles and comprehensive reviews addresses how metabolic processes affect brain development.

A critical step in allowing neurons to communicate efficiently is myelination, a job that is performed by oligodendrocytes in the central nervous system (CNS). Generating myelin is a metabolically costly endeavor, requiring synthesis of lipids and proteins (Tepavcevic, 2021). To accomplish this goal, oligodendrocytes utilize glycolysis to generate ATP, and use lactate to generate lipids (Madeira et al.). Madeira et al. explain the increasingly recognized immune function of oligodendrocytes, work that has been largely accomplished in studies of rodent models. They find that not only do these complex cells wrap and insulate neurons, but they also display immune phenotypes, phagocytosing foreign bodies presenting antigens *via* major histocompatibility class II (MHC II) molecules in cases of neuroinflammation (Madeira et al.). Under conditions of inflammation, such as the demyelinating CNS disease multiple sclerosis (MS), oligodendrocytes redirect their metabolic efforts to generating the immune proteasome, a group of proteins involved in phagocytosis, antigen processing and presentation, becoming immune oligodendrocytes.

Apart from autoimmunity, neuroinflammation is also seen in the context of infection. Early life infections, including TORCH pathogens (toxoplasmosis, other- syphilis and parvovirus B19, rubella, cytomegalovirus CMV, and herpes simplex virus), bacteria, and other emerging pathogens such as Zika virus and SARS-CoV2, can also cause perinatal infection and lead to detrimental brain development through inflammatory pathways. The gut-brain axis has recently come to light as playing an important role in brain homeostasis and response to pathogens (Kirschen et al.). For instance, gut-derived short chain fatty acids can cross the blood brain barrier and activate microglia, the cannonical immune surveillance cells in the CNS, triggering a host inflammatory response through various signaling pathways such as the Jak3/STAT1 pathway (Erny et al., 2015).

Not only are the pathogens themselves responsible for altering metabolic and inflammatory pathways, but the medications used to treat such infections can also influence brain development through gut-brain interactions. Antibiotics used in pregnancy and in the neonatal period have a profound effect on the establishment of the neonatal gut microbiome (Grech et al., 2021). While treatment with antibiotics in cases of infection can of course be life-saving, they have also been shown to alter gut flora composition. Specifically in premature neonates exposed to antibiotics, these neonates demonstrated fewer *Frimicutes* and more *Proteobacteria* species accompanied by altered SCFA profiles compared to neonates not exposed to antibiotics, potentially impacting microglia activity (Dahl et al., 2018; Kirschen et al.).

Antibiotics are one class of drugs that may affect metabolic processes and brain development. On the other hand, recreational substances such as ethanol, marijuana, opioids, and nicotine use can have profound effects on brain structural development, neurogenesis, and synaptic connectivity. Stankovic and Colak explore various metabolic changes occurring in rodent models of prenatal substance exposure. For instance, they find that in rats, ethanol treatment affects one carbon metabolism, increasing cysteine and methionine concentrations in offspring, possibly helping to explain fetal alcohol spectrum disorder, although more work will be needed to fully explain the clinical phenotypes. To address these questions, three dimensional induced pluripotent stem cell-derived human organoids are becoming a more popular tool to model brain development. These brain organoids can be used to examine effects of environmental toxins and drugs on cellular processes, including neurotransmitter biosynthesis, neuronal damage, and regeneration/neurogenesis (Stankovic and Colak).

While small molecules such as ethanol, opioids, and cannabinoids can cross the blood brain barrier and impact brain metabolism directly through their effects on signaling pathways of energy generation and utilization, light is another environmental exposure that may influence brain metabolism and circuitry. Gutierrez-Menendez et al. sought to determine whether photomodulation would affect glucose metabolism and neuronal activity in the prefronal cortex (PFC) and hippocampus. The idea is that light applied directly to the scalp may influence mitochondrial function. They directed red to infrared light non-invasively to the scalps of Wister rats and measured cytochrome c oxidase activity (terminal enzyme in the mitochondrial electron transport chain) and analyzed c-Fos immunohistochemistry (a marker of neuronal activation) in PFC and hippocampus. These investigators found no significant differences between light-exposed and control rats in either of these measures. Regardless, we do know that light has a profound effect on visual cortex development and can positively influence mental health disorders such as depression, impacting brain metabolism likely through indirect signaling from retina to visual cortex to higher level brain regions (Castren et al., 1992; Even et al., 2008).

In summary, brain metabolism can be disrupted or regulated by a host of environmental and intrinsic or genetic mediators, from pathogens, recreational substances and pharmaceuticals to pro-inflammatory molecules and intrinsic cellular machinery. New tools, such as 3-D brain organoids, metabolomics, and gut microbiome profiling, will likely help elucidate the roles that these various mediators play in energy utilization, resource diversion, neurogenesis/gliogenesis, and neuro-regeneration or neurodegeneration.

## Author contributions

GK wrote the initial draft of the manuscript. SG edited the draft. Both authors approved the final version.

## Funding

GK receives internal departmental funding from the Kelly Society at the Johns Hopkins Hospital. SG receives funding from the National Institutes of Health (5R01 AG066912-03).

## Conflict of interest

The authors declare that the research was conducted in the absence of any commercial or financial relationships that could be construed as a potential conflict of interest.

## Publisher's note

All claims expressed in this article are solely those of the authors and do not necessarily represent those of their affiliated organizations, or those of the publisher, the editors and the reviewers. Any product that may be evaluated in this article, or claim that may be made by its manufacturer, is not guaranteed or endorsed by the publisher.

## References

[B1] Castren E. Zafra F. Thoenen H. Lindholm D. (1992). Light regulates expression of brain-derived neurotrophic factor mRNA in rat visual cortex. Proc. Natl. Acad. Sci. USA 89, 9444–9448. 10.1073/pnas.89.20.9444 1409655 PMC50148

[B2] Dahl C. Stigum H. Valeur J. Iszatt N. Lenters V. Peddada S.. (2018). Preterm infants have distinct microbiomes not explained by mode of delivery, breastfeeding duration or antibiotic exposure. Int. J. Epidemiol. 47, 1658–1669. 10.1093/ije/dyy064 29688458

[B3] Erny D. Hrabe de Angelis A. L. Jaitin D. Wieghofer P. Staszewski O. David E.. (2015). Host microbiota constantly control maturation and function of microglia in the CNS. Nat. Neurosci. 18, 965–977. 10.1038/nn.4030 26030851 PMC5528863

[B4] Even C. Schroder C. M. Friedman S. Rouillon F. (2008). Efficacy of light therapy in nonseasonal depression: a systematic review. J. Affect Disord. 108, 11–23. 10.1016/j.jad.2007.09.008 17950467

[B5] Grech A. Collins C. E. Holmes A. Lal R. Duncanson K. Taylor R.. (2021). Maternal exposures and the infant gut microbiome: a systematic review with meta-analysis. Gut Microbes 13, 1–30. 10.1080/19490976.2021.1897210 33978558 PMC8276657

[B6] Tepavcevic V. (2021). Oligodendroglial energy metabolism and (re)myelination. Life (Basel) 11, 238. 10.20944/preprints202102.0277.v1 33805670 PMC7998845

